# Characterization of Amorphous and Co-Amorphous Simvastatin Formulations Prepared by Spray Drying

**DOI:** 10.3390/molecules201219784

**Published:** 2015-12-03

**Authors:** Goedele Craye, Korbinian Löbmann, Holger Grohganz, Thomas Rades, Riikka Laitinen

**Affiliations:** 1 School of Pharmacy, University of Eastern Finland, Yliopistonranta 1C, Kuopio FI-70210, Finland; 2 Department of Pharmaceutical Analysis, University of Ghent, Harelbekestraat 72, Ghent B-9000, Belgium; goedele.craye@ugent.be; 3 Department of Pharmacy, University of Copenhagen, Universitetsparken 2, Copenhagen DK-2100, Denmark; korbinian.loebmann@sund.ku.dk (K.L.); holger.grohganz@sund.ku.dk (H.G.); thomas.rades@sund.ku.dk (T.R.)

**Keywords:** co-amorphous, spray drying, dissolution, solubility, stability, energy-dispersive X-ray spectroscopy

## Abstract

In this study, spray drying from aqueous solutions, using the surface-active agent sodium lauryl sulfate (SLS) as a solubilizer, was explored as a production method for co-amorphous simvastatin–lysine (SVS-LYS) at 1:1 molar mixtures, which previously have been observed to form a co-amorphous mixture upon ball milling. In addition, a spray-dried formulation of SVS without LYS was prepared. Energy-dispersive X-ray spectroscopy (EDS) revealed that SLS coated the SVS and SVS-LYS particles upon spray drying. X-ray powder diffraction (XRPD) and differential scanning calorimetry (DSC) showed that in the spray-dried formulations the remaining crystallinity originated from SLS only. The best dissolution properties and a “spring and parachute” effect were found for SVS spray-dried from a 5% SLS solution without LYS. Despite the presence of at least partially crystalline SLS in the mixtures, all the studied formulations were able to significantly extend the stability of amorphous SVS compared to previous co-amorphous formulations of SVS. The best stability (at least 12 months in dry conditions) was observed when SLS was spray-dried with SVS (and LYS). In conclusion, spray drying of SVS and LYS from aqueous surfactant solutions was able to produce formulations with improved physical stability for amorphous SVS.

## 1. Introduction

Approximately 40% of drug compounds currently on the market and most of the current low molecular weight drug development candidates exhibit poor aqueous solubility [1]. Thus, low water solubility, leading to low and variable oral bioavailability, is an increasing challenge to successful drug development. Several approaches can be employed to enhance the apparent drug solubility and potentially the bioavailability of these challenging drugs, of which amorphous formulations are amongst the most commonly attempted formulation strategies [2,3]. Currently, formulation as solid polymer dispersions is the preferred method to enhance drug dissolution and to stabilize the amorphous form of a drug [3,4]. However, even these systems are often unable to guarantee the long-term stability of amorphous drugs and there are technical challenges with manufacturing and processing of solid dispersions into oral dosage forms [5,6], thus only a few products of this type have reached the market.

Co-amorphous drug systems containing low molecular weight excipients have recently been shown to be a promising approach for stabilization of amorphous drugs and thus are an attractive alternative to the traditional amorphous solid dispersion approach using polymers. The co-amorphous formulations studied so far have been prepared by laboratory-scale methods that are not easily up-scalable, such as quench-cooling, co-milling, and solvent-based methods using organic solvents [7,8,9,10,11,12,13,14,15,16]. However, considering the feasibility of co-amorphous systems for practical applications, these systems need to be prepared with methods that can also be used on an industrial scale and that preferably do not produce hazardous waste. Spray drying is widely used in the pharmaceutical industry for preparation of amorphous materials and also allows continuous production of the material from aqueous solutions [17].

**Figure 1:**
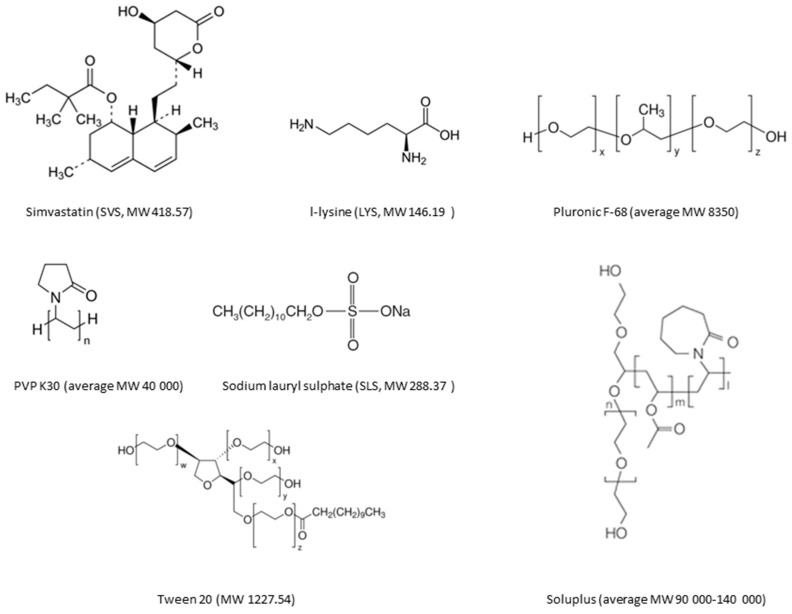
Molecular structures of the studied materials.

In this study, spray drying was explored as a production method for co-amorphous drug/amino acid mixtures containing a poorly water soluble drug, simvastatin (SVS). Previously, SVS has been observed to form a co-amorphous mixture with the amino acid lysine (LYS) upon ball milling [7]. The aim was to perform the spray drying from aqueous solutions, avoiding organic solvents, and to characterize the final powders with respect to their solid-state, dissolution, and stability properties. In general, an aqueous solution is preferred over organic solutions due to toxicity (residues in the final product) and environmental issues associated with organic solvents. Since SVS is a poorly water soluble, neutral drug, an appropriate solubilizer would be required in order to dissolve SVS in water for spray drying from an aqueous solution. The solubilizing capacity of different solubilizers (surfactants and polymers, Figure 1) was first tested in order to find the most effective solubilizer, *i.e*., to minimize the content of the solubilizer in the final product. After finding a suitable solubilizer, the co-amorphous drug/amino acid/solubilizer mixtures were spray dried followed by characterization of the resulting powders. The level of crystallinity in the powders was investigated by X-ray powder diffraction (XRPD), differential scanning calorimetry (DSC) was applied to investigate the thermal properties of the samples, and Fourier-transform infrared spectroscopy (FTIR) provided information about possible interactions between the different components in the mixtures. The powder dissolution and stability properties (storage at 4 °C/0% RH, 40 °C/0% RH and 25 °C/60% RH) of the prepared samples were also investigated.

## 2. Results and Discussion

### 2.1. SVS Solubility in Water in the Presence of Solubilizers

The solubility of SVS in water in combination with different amounts of the solubilizers is shown in Table 1. The solubilizers have different mechanisms of action in solution; *i.e*., in general, the surfactants (Tween 20, SLS, and Pluronic) form micelles that can solubilize hydrophobic drugs. Also the polymers (Soluplus and PVP) may increase the solubility of a drug by forming interactions with the drug molecules without micelle formation (although Soluplus has been observed to form micelles due to its amphiphilic nature) [18]. It should also be noted that the solubilizer concentrations used (*i.e*., >0.5% m/V) were above the critical micelle concentrations (CMCs) of the micelle-forming surfactants [18,19,20,21], which leads to an increase of the drug solubility as a function of the surfactant concentration (Table 1). However, from the table it can be seen that the solubilization capacity of Pluronic and PVP was negligible at every concentration level compared to the other three solubilizers. PVP has been observed to increase the solubility of several drugs as PVP has a tendency to interact with certain molecules and form soluble complexes with them [22,23]. This is due to the large dipole moment of the PVP side groups that strongly interact with other dipoles present. However, for some drugs, such a dipole–dipole interaction is not sufficient to enable complex formation with the drug, in particular if the drug does not have any strong polar groups [18]. Based on the solubility studies with SVS, complex formation between PVP and SVS seems unlikely, since the increase in solubility was less than 3-fold even in the 5% PVP solution. This is in accordance with previous studies [24]. Pluronic is a non-ionic triblock copolymer. Based on the solubility study, the oxyethylene chains of Pluronic were not able to form solubilizing interactions with SVS. In contrast, Soluplus, Tween20, and SLS were found to increase the concentration of dissolved SVS as a function of the solubilizer concentration. This has also been observed previously for SVS with Soluplus [25] and SLS [26].

**Table 1:** Solubility of simvastatin (SVS, µg/mL ± sd.) in water in combination with different amounts (%, m/V) of solubilizers Soluplus, Tween 20, sodium lauryl sulfate (SLS), Pluronic, and Polyvinylpyrrolidone (PVP).

Solubilizer (%, m/V)	Soluplus	Tween 20	SLS	Pluronic	PVP
0	1.74 ± 0.19	1.74 ± 0.19	1.74 ± 0.19	1.74 ± 0.19	1.74 ± 0.19
0.5	27.3 ± 2.5	221.4 ± 33.6	2102 ± 571	2.40 ± 0.39	2.36 ± 0.24
1	53.0 ± 5.6	447 ± 114	5179 ± 2052	2.37 ± 0.45	1.99 ± 0.22
2	112 ± 7	779 ± 4	13599 ± 1351	5.39 ± 0.87	3.93 ± 1.10
5	260 ± 34	2198 ± 641	24338 ± 864	5.57 ± 1.16	4.52 ± 0.83

Tween 20 and SLS were found to be the most effective solubilizers for SVS, with SLS in 5% solution producing the highest solubility for SVS. These are the only surfactants having a long aliphatic hydrocarbon chain in their structure. Thus, it may be that SVS could only be significantly solubilized through hydrophobic interactions between the hydrocarbon chain inside of the micelles and the SVS molecule. Ethanol as a co-solvent led to only marginal increase in solubility (data not shown). Thus, SLS and Tween20 in water were selected for the spray drying experiments.

### 2.2. Preparation of Co-Amorphous Materials by Spray Drying

Three drug–amino acid mixtures including SVS-LYS 1:1 spray-dried from 0.5% SLS, 5% SLS and 5% Tween 20 solutions were prepared (Table 2). To prepare 500 mg SVS-LYS 1:1, 180 mL of 0.5% SLS solution, 16 mL of 5% SLS solution, and 190 mL of 5% Tween20 solution was required. To ensure that SVS was dissolved properly, 20 mL of 5% SLS solution, 200 mL of 0.5% SLS solution, and 200 mL of 5% Tween20 solution were first used instead of the exact values based on the solubility test.

**Table 2:** Summary of the prepared formulations: exact amounts of the components, weight ratios in the final product, spray drying conditions used (T_inlet_, T_outlet_ (°C), pump setting (%)), and the obtained yield.

Spray Drying Conditions						
Formulation	Exact Amount of Every Component	Weight Ratios in Final Product	T_inlet_ (°C)	T_outlet_ (°C)	Pump rate (mL/min)	Yield
SVS-LYS from 5% Tween20	370.6 mg SVS 129.4 mg LYS 200 mL 5% Tween	NA	110	50	4.3-4.7	None
SVS-LYS from 5% SLS	370.6 mg SVS 129.4 mg LYS 20 mL 5% SLS	28.51 % SVS 9.95% LYS 61.54% SLS	150	65	4.7	Very low
SVS-LYS from 5% SLS	370.6 mg SVS 129.4 mg LYS 20 mL 5% SLS	24.71 % SVS 8.63% LYS 66.67% SLS	110	50	4.3-4.7	Low (12%)
SVS-LYS from 5% SLS	370.6 mg SVS 129.4 mg LYS 16 mL 5% SLS	28.51 % SVS 9.95% LYS 61.54% SLS	100	45	3.9	Satisfactory (24%)
SVS-LYS from 0.5% SLS	370.6 mg SVS 129.4 mg LYS 200 mL 0.5% SLS	NA	110	50	4.3-4.7	Satisfactory (23%)
SVS from 5% SLS	370.6 mg SVS 129.4 mg LYS 20 mL 5% SLS	31.70 % SVS 68.30% SLS	100	45	3.9	Low (15%)

NA: not applicable.

It was observed that no dry powder could be produced by spray drying SVS-LYS from 5% Tween20 aqueous solution, which was not surprising considering that Tween20 is a viscous liquid at room temperature. Using SLS solutions, different formulation (0.5% or 5% SLS solution) and processing conditions (T_inlet_, and T_outlet_ which was an outcome of T_inlet_ and the pump rate) were applied (Table 2). As can be seen from Table 2, higher T_inlet_, and consequently T_outlet_, temperatures produced low yields, whereas lower temperatures improved the results when 5% SLS was used. Using more dilute SLS (0.5%) improved the yield at inlet 110 °C, but reducing the volume of the 5% SLS solution and decreasing the inlet to 100 °C improved the yield slightly further. Thus, the best spay drying conditions were the ones where T_inlet_ was 100 °C, T_outlet_ was 45 °C, pump rate was 3.9 mL/min when using a 5% SLS solution. These conditions were used to produce spray-dried SVS-LYS for further studies.

Spray drying of drugs, having a relatively low glass transition temperature (T_g_), such as SVS, is difficult since often stable amorphous products in the form of a free flowing powder cannot be obtained [21]. T_outlet_ being above the T_g_ values of the products may be the reason for the relatively low yields, making the powder stick to the surfaces of the spray dryer [27]. The solid state form (amorphous/crystalline) and the T_g_-values of the powders obtained were subsequently studied.

### 2.3. Characterization of the Spray-Dried Materials

#### 2.3.1. X-ray Powder Diffraction (XRPD)

**Figure 2:**
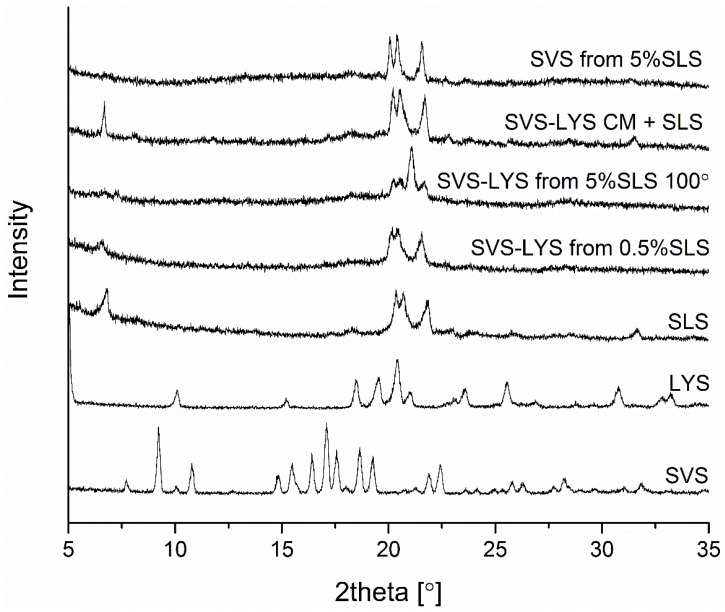
XRPD diffractograms of SVS, LYS, SLS, and the spray-dried formulations of SVS-LYS from 0.5% SLS, SVS-LYS from 5% SLS, SVS from 0.5% SLS, and SVS-LYS CM + SLS.

The XRPD diffractograms of crystalline SLS and the spray-dried formulations are shown in Figure 2. In addition, the diffractogram of SVS-LYS CM (cryo-milled) physically mixed with SLS is shown (SVS-LYS CM + SLS). The XRPD-analysis of the powder samples (Figure 2) revealed that pure SLS showed characteristic diffraction peaks [28] at 2θ values of 6.8°, 18.2°, 20.3°, 20.7°, 21.8°, and 31.7° and that the remaining crystallinity in the spray-dried samples originated from SLS, *i.e*., no SVS and/or LYS diffraction peaks [7] were observed. However, some changes in SLS crystallinity in the spray-dried samples might have occurred. The SLS peak at a 2θ-value of 6.8° had disappeared completely in SVS-LYS spray-dried from 5% SLS and in SVS spray-dried from 5% SLS. The SLS peak at a 2θ-value of 31.7° had disappeared in all spray-dried samples. The major diffractions at a 2θ-value of approx. 21° had remained unchanged in SVS-LYS spray-dried from 0.5% SLS and in SVS spray-dried from 5% SLS. However, the shape of this diffraction feature had changed in SVS-LYS from 5% SLS, indicating a possible change in the crystal structure of SLS.

#### 2.3.2. Particle Surface Characteristics

**Figure 3:**
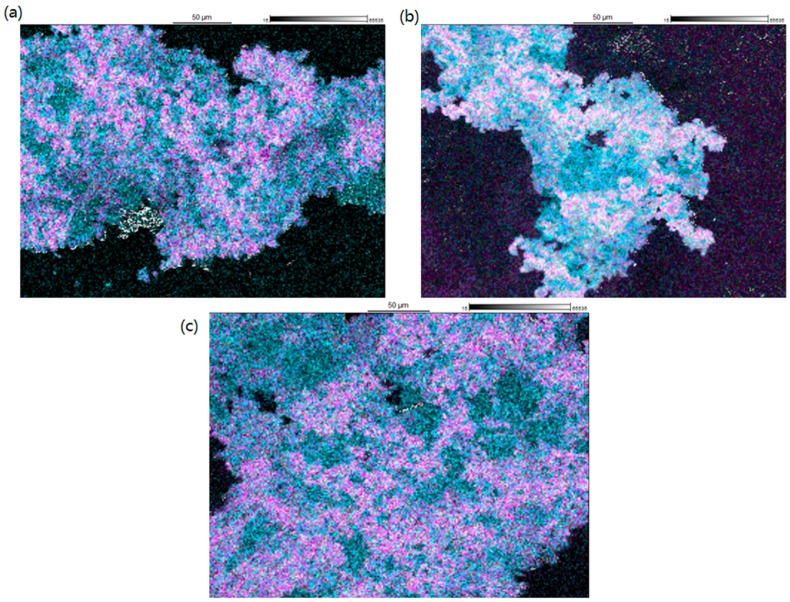
Distribution of Na (•) and S (•) on the spray-dried particle surfaces measured by EDS: (**a**) SVS-LYS from 5% SLS; (**b**) SVS-LYS from 0.5% SLS; and (**c**) SVS from 5% SLS.

Elemental analysis of the spray-dried particle surfaces was conducted with a scanning electron microscope (SEM) equipped with an energy-dispersive X-ray (EDS) detector. As can be seen from Figure 1, SVS consists only of carbon (C), oxygen (O), and hydrogen (H). In addition to these elements, LYS also contains nitrogen (N). SLS in turn contains C, O, H, sulfur (S), and sodium (Na). Based on this, SLS could be differentiated from the other components in the particles. Since SLS is a surface-active agent, it could be anticipated that it would accumulate on the droplet surfaces in the atomized feed solution and thus on dry particle surfaces during the spray drying process [29]. This has also been observed when the surfactant is present in the spray drying solution above its critical micelle concentration (CMC) [30], as is the case in the SLS solutions used in this study. Although forming micelles above the CMC, the gas–liquid interface of the droplets will be saturated with the surfactant monomers [31], which then accumulate on the dry particle surfaces. The EDS measurements revealed that SLS in fact coated the particles as Na and S were concentrated on the particle surfaces (Figure 3). However, there are differences in the relative amounts of Na and S on the surface when comparing the different formulations. When SVS-LYS was spray-dried from more dilute SLS solution (*i.e*., 0.5%), there seems to be less Na and S (*i.e*., less SLS) on the particle surface compared to the formulations dried from the 5% SLS solution (even though the absolute amount of SLS in the final powder was the same).

#### 2.3.3. Differential Scanning Calorimetry (DSC)

The thermal properties of the prepared samples were investigated using DSC. Table 3 shows the melting (T_m_), glass transition (T_g_), and recrystallization temperatures (T_rc_) of all samples.

**Table 3:** Melting temperature (T_m_), glass transition (T_g_), and recrystallization temperature (T_rc_) of the pure starting materials compared to the prepared samples.

Material	T_g_ (°C)	T_rc_ (°C)	T_m_ SVS (°C)	T_m_ LYS (°C)	T_m_ SLS (°C)
SVS ^a^	29.0 ± 0.6	NA	139.9 ± 0.2	NA	NA
LYS ^a^	68 ^b^	NA	NA	212.4 ± 0.5 ^a^	NA
SLS	ND	NA	NA	NA	194.3 ± 0.5 ^c^
SVS-LYS CM ^a^	33.2 ± 0.9	107.0 ± 1.2	134.1 ± 0.1	199.9 ± 3.2	NA
SVS-LYS from 0.5% SLS	23.6 ± 5.7	73.1 ± 0.8	143.7 ± 0.2	ND	162.9 ± 0.4
SVS-LYS from 5% SLS	26.2 ± 3.8	68.6 ± 9.6	140.6 ±1.0	ND	157.6 ± 0.4
SVS from 5% SLS	13.7 ± 0.6	ND	ND	NA	182.7 ± 0.6

^a^: Values from [7]; ^b^: Value from [32]; ^c^: Another endothem was seen at 103.1 ± 7.2; NA: not applicable; ND: not detected.

From Table 3 it can be seen that two endothermic peaks were observed in the thermogram of pure SLS at 103.13 °C (loss of water) and 194.25°C (melting), which is in agreement with literature data [33,34,35,36]. The lower endotherm of SLS was observed in all the spray-dried samples. In addition, a recrystallization peak was observed at a temperature range of 68–75°C for the spray-dried samples containing LYS, which was in accordance with the T_rc_ previously measured for amorphous SVS [7]. Previously, it has been found that the T_rc_ was increased to 107 °C in the case of SVS-LYS CM, pointing towards a stabilizing effect by formation of the co-amorphous mixture with LYS [7]. A similar phenomenon, however, was not observed when SLS was included in the spray-dried formulations with SVS and LYS. After recrystallization, an endothermic melting peak of SVS was observed at approx. 140 °C in these samples. In contrast, neither a recrystallization peak nor a melting peak of SVS were observed in the thermogram of SVS from 5% SLS, which is indicative of a higher stability against recrystallization for this formulation [37,38]. Instead, an SLS melting peak was observed, but at approximately 10 degrees lower than in pure SLS. In the formulations containing LYS, the SLS melting peak was very small and observed at temperatures 30 degrees lower than in pure SLS. This may be an indication of changes in the crystalline character of SLS and/or for partial miscibility of SLS with SVS and LYS. Furthermore, one single T_g_ was observed for all spray-dried samples, indicating formation of a homogenous amorphous phase [39,40]. In general, the observed T_g_ was somewhat lower than for pure amorphous SVS (Table 1) and amorphous SVS-LYS (determined previously to be 33.2 °C [7]). This indicated that SLS, which has a low melting point and thus a low T_g_ [41], was partially miscible with the amorphous phase formed by SVS and LYS and acted as a plasticizer in the mixtures. SLS seemed to have the largest plasticizing effect on SVS from 5% SLS, as the T_g_ of this mixture was found to be lowest.

After these measurements SVS-LYS from 0.5% SLS was not characterized further due to a long spray drying time as a consequence of the large volume of 0.5% SLS solution required for dissolving SVS.

#### 2.3.4. FTIR

**Figure 4:**
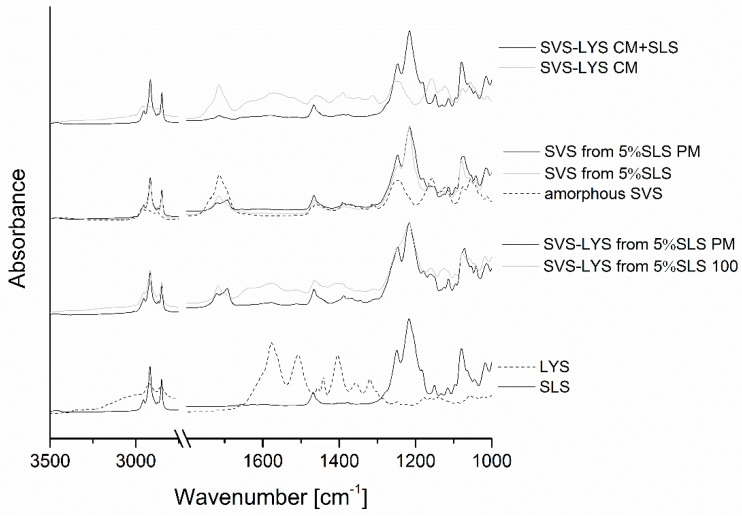
FTIR spectra of the prepared samples and corresponding physical mixtures (PMs). In addition, the spectra of amorphous SVS (prepared by CM) and SVS-LYS CM are shown for comparison.

After the spray drying process with SLS, peak broadening and peak shifts were seen at the SVS ester C=O stretch absorption region from 1695 cm^−1^ to 1715 cm^−1^ and from 3550 to 3450 cm^−1^ (SVS OH-region) when compared to crystalline SVS (Figure 4). These shifts were also observed in amorphous SVS and co-amorphous SVS-LYS and they can be considered to originate from the amorphization of SVS [7]. The most intense band observed in the spectra of the spray-dried samples was the SO_3_ asymmetric vibrational feature (ν_as_(SO_3_)) of SLS, which in general is observed as a double peak in crystalline, ordered SLS [42,43,44]. In the spectrum of crystalline SLS, the SLS SO_3_ asymmetric stretching is a large duplet at 1244 cm^−1^ and 1217 cm^−1^ with a small shoulder at 1180 cm^−1^ (Figure 4). It covers the SVS-related absorption feature at 1245 cm^−1^ (seen in amorphous SVS and SVS-LYS CM). This SLS SO_3_ -absorption feature was broadened in SVS from 5% SLS but merged to one large peak between 1170 cm^−1^ and 1300 cm^−1^ in SVS-LYS from 5% SLS, indicating disruption of the structural order of SLS. In addition, the shoulder peak at 1180 cm^−1^ was not seen in both types of spray-dried mixtures (with or without LYS), which may be indicative of interactions of this group with SVS. Peak broadening of other SLS-related features was observed in the spray-dried samples. The FTIR spectra of SVS-LYS from 5% SLS and the corresponding physical mixture (PM) showed two peaks at 1580 cm^−1^ and 1512 cm^−1^ (amide I and amide II bands of LYS). These peaks were less sharp in SVS-LYS from 5% SLS than in the corresponding PM, but they did not merge into one large peak upon spray drying, in contrast to what was observed in co-amorphous SVS-LYS CM [7]. The aliphatic chain vibrations of LYS, which shifted to lower wavelengths upon formation of co-amorphous SVS−LYS (*i.e*., to 1350 cm^−1^ and 1312 cm^−1^) [7], can be seen at even lower wavelengths in SVS-LYS from 5% SLS (*i.e*., at 1348 cm^−1^ and 1309 cm^−1^). In addition, the absorption band at 1390 cm^−1^ (originating from LYS) appears less sharp in the spectra of SVS-LYS from 5% SLS than in the spectrum of SVS-LYS CM. These changes indicate that in SVS-LYS from 5% SLS, SLS may have disturbed formation of the weak interactions between SVS and LYS, which formed in co-amorphous SVS-LYS. This is also in accordance with the DSC results, which showed that an increase in T_rc_ in SVS-LYS CM compared to amorphous SVS was not observed when SLS was incorporated in the formulation.

#### 2.3.5. Dissolution Properties

**Figure 5:**
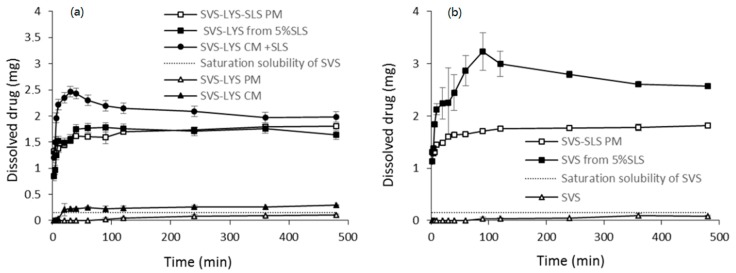
The cumulative amounts of SVS dissolved for (**a**) SVS-LYS formulations and (**b**) SVS formulations in phosphate buffer (pH 7.2). The point of 24 h is not shown, since it was not statistically different from the value obtained at 480 min. The data for SVS, SVS-LYS PM, and SVS-LYS CM formulations is taken from [45].

In addition to the spray-dried formulations, the dissolution profiles of the corresponding physical mixtures and SVS-LYS CM + SLS were determined in phosphate buffer (pH 7.2) (Figure 5). This was done to have a fully crystalline reference (PM), a reference with amorphous SVS-LYS (CM) physically mixed with SLS, and a mixture where SVS-LYS and SLS were all processed together.

The saturation dissolvable amount of crystalline SVS (without SLS) in these conditions has been previously determined to be 0.15 mg [45]. The same study showed that amorphization of SVS alone did not improve the dissolution of SVS. In contrast, combining SVS with LYS in a co-amorphous formulation (SVS-LYS CM) improved the dissolution significantly (the concentrations were three times higher with SVS-LYS CM than with SVS-LYS PM). LYS in a simple crystalline physical mixture with SVS was not able to enhance the dissolution compared to crystalline SVS (see Figure 5a) [45]. In Figure 5 it can also be seen that SLS had a significant solubilizing effect (approx. 10-fold) on crystalline SVS, as the maximum dissolved amount of SVS was 1.8 mg in the case of PMs. However, when the amorphous SVS-LYS CM was physically mixed with SLS, the dissolution increased even further (this combination had the best dissolution properties amongst the ternary formulations). The spray-dried formulation (SVS-LYS from 5%SLS) had dissolution properties almost identical to SVS-LYS-SLS PM (not significantly different (*p* > 0.05) from the corresponding PM). However, in the case of SVS-LYS from 5% SLS (Figure 5a), the maximum released amount of SVS (1.8 mg) was already reached after 90 min while the maximum released amount of SVS (1.8 mg) for SVS-LYS-5% SLS PM was seen later, after approximately 360 min. It can be stated that the dissolution curve of SVS-LYS from 5% SLS was not significantly different (*p* > 0.05) from the corresponding PM. Thus, the amorphous form in this case did not enhance the dissolution further. However, the dissolution of SVS-LYS CM + SLS was significantly faster (*p* < 0.05) than that of SVS-LYS spay dried from 5% SLS. In addition this formulation enabled higher concentrations of SVS than the others. The maximum released amount of SVS (2.46 mg) in the case of SVS-LYS CM + SLS was already seen after 30 min. This formulation was thus able to provide a “spring and parachute” effect [46], *i.e*., rapid supersaturation (peak concentration at 30 min), which was maintained until approx. 240 min. Compared to the dissolution properties of SVS-LYS CM determined previously in similar conditions (shown in Figure 5a) [45], the amount of dissolved SVS was approx. 10-fold with SVS-LYS CM + SLS. In addition, the peak concentration was achieved much faster with SVS-LYS CM + SLS (*i.e*., 30 min *vs.* 480 min).

In contrast, SVS-SLS PM (Figure 5b) had similar dissolution properties to SVS-LYS from 5%SLS and SVS-LYS-SLS PM. Thus, it seems that LYS does not have a positive effect on dissolution in a completely crystalline mixture (SVS-LYS-SLS PM *vs.* SVS-SLS PM) or when spray dried as a ternary combination (SVS-LYS from 5%SLS *vs.* SVS-LYS-SLS PM *vs.* SVS-SLS PM). LYS is able to improve the dissolution of SVS only when combined with SVS as a co-amorphous binary formulation (SVS-LYS CM or SVS-LYS CM + SLS).

An even more enhanced “spring and parachute” effect than that seen with SVS-LYS CM + SLS was observed for SVS spray-dried from 5% SLS (Figure 5b), with the maximum dissolved amount of SVS (3.23 mg) reached after 90 min. This was almost twice the maximum dissolved amount of the PM mixture (Figure 5). The supersaturated state generated by the amorphous form was maintained throughout the experiment, as the dissolution from the spray-dried formulation was significantly enhanced (*p* < 0.05) compared to that of the corresponding PM and to the second best formulation, *i.e*., SVS-LYS CM + SLS. The results thus indicate that the dissolution properties of SVS from the co-amorphous SVS-LYS can be improved by physically mixing with SLS but co-processing of these three components together was not beneficial. Instead, the best dissolution properties for SVS dissolution were achieved by spray drying SVS with SLS.

#### 2.3.6. Physical Stability

The physical stability of the formulations was investigated after storage under different conditions *i.e*., 4 °C/0% RH, 40 °C/0% RH, and 25 °C/60% RH. The stability of the spray-dried formulations was compared with SVS-LYS CM + SLS. This was done in order to investigate whether there was any difference in the stability if SLS is added simply as a crystalline component to the amorphous SVS-LYS CM in contrast to all components being processed together.

**Figure 6:**
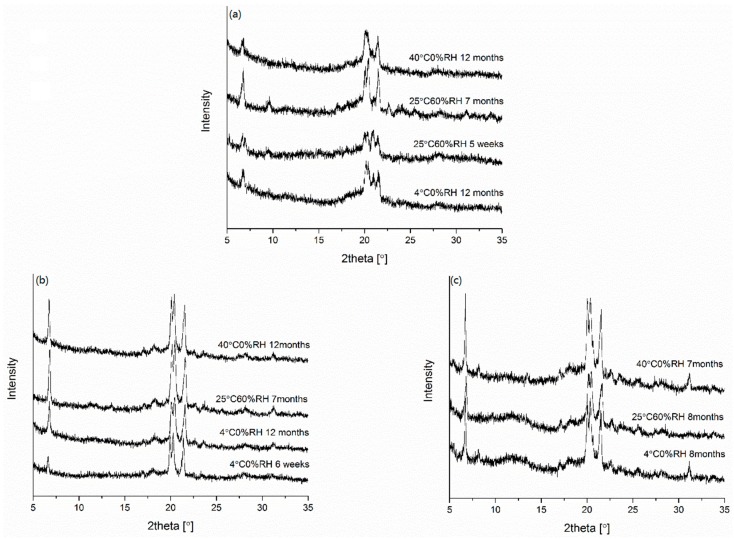
X-ray diffractograms of the spray-dried powders after storage under different conditions: (**a**) SVS-LYS spay dried from 5% SLS; (**b**) SVS spray-dried from 5% SLS; (**c**) SVS-LYS CM + SLS.

Interestingly, even though the formulations were only partially amorphous initially (remaining SLS crystallinity) the changes observed as a function of time in different conditions were relatively small (Figure 6). The SVS-LYS formulation spray-dried from 5% SLS (Figure 6a), was found to be stable at 4 °C/0% RH and 40 °C/0% RH for at least one year. Compared to the freshly prepared sample (Figure 2), the only changes occurring under these conditions were changes in SLS-related diffractions. The SLS-related peak at 6.8° 2θ appeared after five weeks of storage at 4 °C/0% RH and after two months at 40 °C/0% RH. No peaks related to crystalline SVS or LYS appeared during the storage. When stored at 25 °C/60% RH, peaks appeared also at 9.5° (at five weeks) and at 22.5° 2θ (at eight months) which can be assigned to SVS [8]. Interestingly, the peak shapes of the four major SLS-diffractions (between 20° and 22° 2θ) changed during storage, depending on the conditions. At 4 °C/0% RH, the relative height of the third peak decreased and at 25 °C/60% RH and 40 °C/0% RH it disappeared completely. Thus, only samples stored at 25 °C/60% RH for seven months and at 40 °C/0% RH for 12 months showed a diffraction pattern that resembles the diffraction pattern of pure crystalline SLS.

In the case of SVS spray dried from 5% SLS (Figure 6c), the observable changes in all conditions were related to SLS diffractions. At 4 °C/0% RH, the SLS peaks at 6.8° and 18.2° 2θ appeared after three weeks of storage but no other changes occurred in the diffractogram during a one-year storage period (the major diffractions were more or less unchanged already in the fresh sample when compared to crystalline SLS). At 25 °C/60% RH and 40 °C/0% RH, these peaks appeared already after two weeks of storage. In addition, a SLS peak at 31.7° 2θ was seen at all storage conditions. However, no other changes were observed in the diffractograms until one year of storage (40 °C/0% RH) and seven months of storage (25 °C/60% RH).

In the case of SVS-LYS CM + SLS, two new peaks appeared during the storage period (at seven months at 40 °C/0% RH and at eight months in the other conditions). As crystalline SLS was physically mixed with co-amorphous SVS-LYS, the changes were related to recrystallization of this co-amorphous mixture. The peaks appearing at 7.8° 2θ (at eight months in 4 °C/0% RH and seven months in 40 °C/0% RH) and 17.2° (at eight months in 4 °C/0% RH and 25 °C/60% RH , and at seven months in 40 °C/0% RH) could be attributed to recrystallization of SVS from the mixture [7,8].

**Figure 7:**
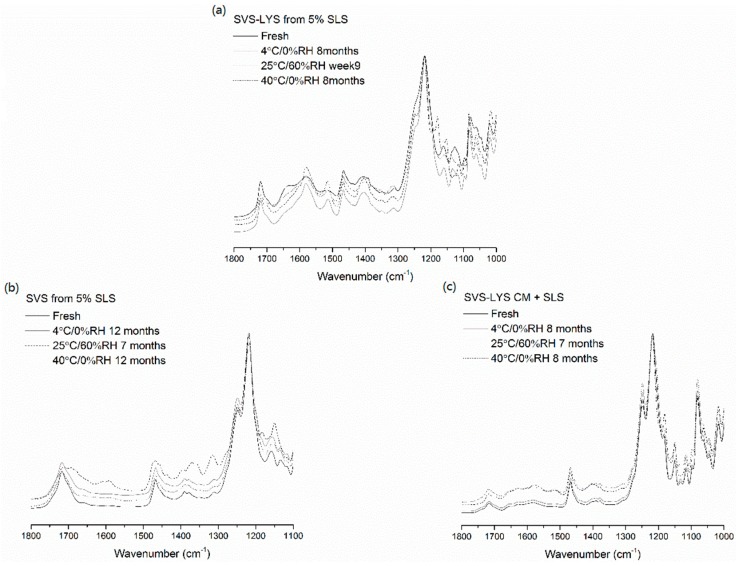
FTIR-spectra of the spray-dried powders after storage under different conditions: (**a**) SVS-LYS spray-dried from 5% SLS; (**b**) SVS spray-dried from 5% SLS; (**c**) SVS spray-dried from 5% SLS and SVS-LYS CM + 5% SLS.

The FTIR-spectra of the stored samples are shown in Figure 7. In the case of SVS-LYS spray-dried from 5% SLS, the SO_3_ asymmetric vibrational double peak of SLS that was merged upon spray drying (at 1170 cm^−1^–1300 cm^−1^) was again split to a double peak during storage. The peak splitting was visible after eight months when stored at 4 °C/0%RH and 40 °C/0%RH. Instead, at storage conditions of 25 °C/60%RH the peak splitting was seen at nine weeks. In addition, the shoulder peak at 1180 cm^−1^ (from SLS), which disappeared after spray drying, reappeared in its original position at week one in the FTIR spectrum of the sample stored at 25 °C/60%RH. In the other storage conditions, this peak did not reappear. However, the SVS ester C=O stretch absorption band, which shifted from 1695 cm^−1^ to 1715 cm^−1^ upon amorphization, was unchanged at all storage conditions. Instead, the amide I and amide II bands of LYS at 1580 cm^−1^ and 1512 cm^−1^ became sharper upon storage at all conditions.

In SVS spray-dried from 5% SLS, no changes were observed in the SO_3_ asymmetric vibrational double peak of SLS compared to the fresh sample during storage. The shoulder peak at 1180 cm^−1^ also reappeared in the FTIR spectrum of the sample stored at 25 °C/60%RH at seven months. However, the SVS ester C=O stretch absorption band at 1715 cm^−1^ was unchanged in all storage conditions (except broadened in 25 °C/60%RH). The spectrum of the sample stored at 25 °C/60%RH showed a rise in the baseline level, which probably indicates moisture absorption into the sample.

In SVS-LYS CM + SLS the SLS-shoulder peak at 1180 cm^−1^ became higher at 25 °C/60%RH at seven months. In addition, in these conditions the SVS ester C=O stretch absorption band at 1715 cm^−1^ was slightly split, indicating recrystallization of SVS.

In a previous study with the co-amorphous SVS-LYS CM formulation [7], it was found that this formulation was still X-ray amorphous after five months of storage at 4 °C/0% RH. At an elevated temperature (40 °C/0%RH), the mixture showed signs of recrystallization within three months and at elevated humidity (25 °C/60% RH) at day 56. Compared to this, all the formulations prepared in the current study were able to significantly extend the stability of amorphous SVS, which was surprising due to the fact that at least partially crystalline excipient (SLS) was present in the formulations. In addition, surfactants cannot be considered as stabilizing excipients for amorphous materials in general [47]. It has been found that surfactants can increase the crystal growth rate of amorphous celecoxib if they are miscible with the drug [41]. However, SLS, being immiscible with celecoxib, was found to have very little impact on the growth rate of celecoxib crystals. When the miscibility was improved by adding polyvinylpyrrolidone (PVP) to form a ternary dispersion of celecoxib, SLS was then found to increase the growth rate of celecoxib compared to the corresponding binary celecoxib–PVP blends. In the current study, the best stabilizing effect was found (at least 12 months in dry conditions), when SLS was spray-dried with SVS (and LYS) instead of being physically mixed with the co-amorphous SVS-LYS. Since SLS was only partially miscible with SVS (*i.e*., lowered the T_g_ of the mixture slightly), and thus remained mainly in the crystalline phase in the studied mixtures, it seems less likely that miscibility of SLS had an impact on the stability of SVS. Instead, the stabilizing effect could be due to a protective coating produced by SLS upon spray drying, which provided physical separation of the amorphous material (Figure 3) [48]. Furthermore, the hygroscopicity of SLS has been found to be low below RH values of 75%, after which it shows deliquescence [49]. Thus, SLS might protect the amorphous content form ambient moisture below this RH value. The results also indicated that in this case incorporating LYS as a co-amorphous former did not bring significant stability or dissolution advantages when compared to formulations containing only amorphous SVS.

## 3. Experimental Section

### 3.1. Materials

Simvastatin (SVS) and the amino acid lysine (LYS) were supplied by Hangzou Dayangchem Co., Ltd. (Hangzou, China). The solubilizers polysorbate 20 (Tween20), polyvinylpyrrolidone K-30 (PVP), and Pluronic F-68 were obtained from Sigma-Aldrich Chemie GmbH (Steinheim, Germany). Sodium lauryl sulfate (SLS) and Soluplus were provided by YA-Kemia Oy (Helsinki, Finland) and BASF SE (Ludwigshafen, Germany) respectively. The compounds were used as received.

### 3.2. Solubility Test

The solubility of SVS in water in combination with five different solubilizers was measured. Two polymers were used (*i.e*., PVP and Soluplus) and three surfactants (*i.e*., Pluronic, SLS, and Tween20). Four concentrations (m/V) (5%, 2%, 1%, and 0.5%) of each solubilizer were prepared and an excess of the drug was added to 10 mL of every solution. This was done in triplicate for every solubilizer concentration. The samples were put on a shaker at room temperature for three days, after which the solutions were filtered through a 0.21 µm pore size membrane filter and analyzed with high-performance liquid chromatography (HPLC) as described below.

In addition, the solubility was determined in a water–ethanol (20:80) mixture with potential solubilizers (1% and 2.5% SLS, and 2.5% and 5% Soluplus) in order to investigate the influence of ethanol on the solubility, since the use of ethanol could reduce the amount of solubilizer. Twenty percent ethanol was applied since this is the highest concentration that can be safely used in the spray dryer device used in this study.

### 3.3. Preparation of the Materials

The SVS:LYS molar ratio used is this study was 1:1. The selection of solubilizer and the amount needed to dissolve the drug were based on the solubility study. A predetermined amount of the solubilizer was dissolved in water and subsequently SVS and LYS were added to the solution. The solution was mixed until the components were dissolved.

Spray drying was performed with a Büchi mini spray dryer (Model B-191, Büchi Labortechnik AG, Flawil, Switzerland). The following conditions were used: inlet temperature was 100 °C, outlet temperature was adjusted to 45 °C by adjusting the pump to 15% to give a flow rate of 3.9 mL/min, (see Table 2). Aspirator setting was always 100% and gas flow rate 600 L/h. The spray drying process of SVS-LYS from 5% SLS mixture with an inlet temperature of 110 °C and 150 °C was also investigated and in this case the pump setting and the flow rate varied between 18% and 20% and 4.3–4.7 mL/min, respectively, producing outlet temperatures between 50 and 65 °C.

SVS-LYS (molar ratio of 1:1) was also prepared by cryo-milling (CM) before physically mixing with a surfactant (the amount corresponding to what was present in the final product spray-dried from 5% SLS solution). The two milling chambers were filled with 500 mg SVS-LYS mixture and two 15 mm stainless steel balls. Milling was performed at 30 Hz in an oscillatory ball mill (Mixer Mill MM 400, Retch GmbH & Co., Haan, Germany). The total milling time was 60 min. Prior to milling and after every 10 min, the milling chambers were immersed in liquid nitrogen for 2 min to ensure cryogenic conditions. When the milling process was finished, the milling chambers were placed in a desiccator over silica. After they reached room temperature, the products were weighed and mixed with the correct amount of surfactant in a mortar.

Physical mixtures (PMs) of the unprocessed starting materials were prepared by mixing with pestle in a mortar.

### 3.4. X-ray Powder Diffraction (XRPD)

XRPD was performed using Bruker D8 Discover X-ray diffractometer (Karlsruhe, Germany) applying Cu Kα radiation (λ = 1.54 Å). Scanning of the samples was carried out between 5° and 35° 2θ with a step size of 0.011° and a scan speed of 0.1 s/step. An acceleration voltage of 40 kV and current of 40 mA were used. The data was collected by DIFFRAC.V3 program.

### 3.5. Elemental Analysis of Spray-Dried Particle Surfaces

The spray-dried particles were analyzed with a Zeiss Sigma HD VP scanning electron microscope (SEM, Carl Zeiss Microscopy, Jena, Germany) equipped with two energy-dispersive X-ray (EDS) detectors (60 mm^2^, Thermo Noran, Thermo Fisher Scientific, Waltham, MA, USA). The particles were fixed on an aluminum sample stub by using a carbon adhesive and subsequently scanned with an acceleration voltage of 6 keV under 5 Pa nitrogen atmosphere (except 10 Pa for SVS from 5% SLS and SVS-LY from 0.5% SLS).

### 3.6. Differential Scanning Calorimetry (DSC)

The DSC analysis was performed using a Mettler Toledo DSC823e (Schwerzenbach, Switzerland) attached with a Julabo FT 900 Cooler. The DSC thermograms of the samples were obtained under a nitrogen gas flow of 50 mL/min. Aluminum pans (40 µL) were filled with sample powder (4–8 mg) and heated at a rate of 10 K/min from −50 °C to 220 °C. Crystalline SLS was heated from 25 °C to 220 °C at 10 K/min and held for 5 min at the final temperature. Afterwards, the melt was immediately cooled to −50 °C, where it was held for 15 min. Thereafter, it was heated again from −50 °C to 250 °C.

STARe software was used to determine the glass transition temperatures (T_g_, midpoint), melting temperatures (T_m_, onset), and recrystallization temperatures (T_rc_, onset) of the samples. These were calculated as the mean of three independent measurements.

### 3.7. Fourier-Transform Infrared Spectroscopy (FTIR)

Infrared spectroscopy was performed with a FTIR Thermo Nicolet Nexus 8700 FT-IR (Thermo Scientific, Madison, WI, USA) using an ATR (attenuated total reflectance) accessory. Thermo Scientific OMNIC software (version 6.0a, Thermo Scientific) was applied to collect the samples. The spectra (64 scans) were recorded in a wave number range of 550 to 4000 cm^−1^ with a resolution of 4 cm^−1^.

### 3.8. Dissolution Testing

Dissolution testing was performed using a Distek dissolution system 2100 C (North Brunswick, NJ, USA) with a paddle rotation speed of 50 rpm and temperature of 37 °C. A powder amount equivalent to 20 mg of the drug was placed on the bottom of the dissolution chamber. The chamber was subsequently filled with 500 mL of the preheated dissolution medium. The dissolution medium was a phosphate buffer with pH 7.2 (USP). All formulations were measured in triplicate.

The dissolution profiles were measured over a period of 24 h. At predetermined intervals (2, 4, 6, 10, 20, 30, 40, 60, 90, 120, 240, 360, 480, and 1440 min), 5-mL aliquots were taken from the chamber and immediately replaced with the same volume of phosphate buffer solution (pH 7.2). After filtering the samples through a 0.21 µm membrane filter, the filtrates were immediately diluted with acetonitrile (ACN, VWR International S.A.S., Fontenay-Sous Bois, France) to correspond to the mobile phase composition (see below). Further dilution, if necessary, was done by 70/30 ACN/H_2_O. Samples were analyzed with high-performance liquid chromatography (HPLC) as described below. Single-factor ANOVA analysis was performed to investigate whether the dissolution profiles of two formulations were statistically different (95% confidence level).

### 3.9. High-Performance Liquid Chromatography (HPLC)

Quantitative determination of the drug concentrations was performed using a Gilson HPLC analysis system with UV-VIS 151 detector (Gilson, Middleton, WI, USA), 234 auto-injector (Gilson, Villiers le Bel, France), 321 pump (Gilson, Villiers le Bel, France), system interface module (Gilson), and Unipoint TM LC system version 3.01 software (Gilson). A Phenomenex Gemini-NX 5 µm C18 110 Å (250 × 4.6 mm) column and SecurityGuard precolumn (Phenomenex Inc., Torrance, CA, USA) were used.

The drug concentrations were determined using a mobile phase consisting of 70% ACN, 30% ultrapure water, and 0.1% trifluoroacetic acid (TFA, Sigma-Aldrich, St. Louis, MO, USA). The mobile phase flow rate was 1.2 mL/min and the detection wavelength was 238 nm. Standard solutions with concentrations of 0.5, 1, 5, 10, 25, 50, and 100 µg/mL of the drug were prepared in 70/30 ACN/H_2_O.

### 3.10. Stability Study

The formulations selected for the stability study were stored at 4 °C/0% relative humidity (RH), room temperature (25 °C)/60%RH, and 40 °C/0% RH. Phosphorous pentoxide was used to achieve the RH of 0% while the RH of 60% was obtained with a saturated NaBr solution. The samples were analyzed regularly with XRPD and FTIR to detect solid-state changes occurring in the samples.

## 4. Conclusions

In this study, co-amorphous drug–amino acid mixtures were spray-dried form aqueous solutions for the first time by using a surface-active agent sodium lauryl sulfate (SLS) as a solubilizer for the poorly water-soluble drug simvastatin (SVS). SVS and lysine (LYS) were dissolved at a 1:1 molar ratio in 0.5% or 5% SLS water solutions, which were then spray-dried to obtain the formulations of SVS-LYS from 0.5% SLS and SVS-LYS from 5% SLS. In addition, a spray-dried formulation without LYS (SVS from 5% SLS) and a co-milled SVS-LYS reference, physically mixed with SLS, were prepared.

Elemental analysis by energy-dispersive X-ray spectroscopy (EDS) revealed that SLS coated the particles that were formed upon spray drying. Solid-state analysis by X-ray powder diffraction (XRPD) and differential scanning calorimetry (DSC) revealed that in all the spray-dried formulations the remaining crystallinity originated from SLS only. However, the crystalline structure of SLS was modified in these mixtures, as revealed by changes in the shape of the XRPD diffraction peaks of SLS, melting behavior of SLS, and the SO_3_ asymmetric vibrational feature of SLS in the Fourier-transform infrared (FTIR) spectra of the samples.

SVS from 5% SLS formulation showed the best dissolution properties in pH 7.2, as a “spring and parachute” effect was observed. The maximum dissolved amount of SVS, which was almost twice the maximum dissolved amount of the corresponding physical mixture, was reached after 90 min.

Despite the presence of at least partially crystalline SLS in the mixtures, all the studied formulations were able to significantly extend the stability of amorphous SVS compared to what has previously been seen with co-amorphous formulations of SVS. The best stabilizing effect (at least 12 months in dry conditions) was found when SLS was spray-dried with SVS (and LYS) instead of physically mixing it with co-amorphous SVS-LYS. The stabilizing effect of SLS could be due to physical separation of the amorphous material, as SLS was found to coat the spray-dried particles. In addition, SLS might protect the amorphous content form ambient moisture.

In conclusion, spray drying of SVS (and LYS) from aqueous surfactant solutions was able to produce formulations with improved physical stability for amorphous SVS. This study showed that particle coating with a surface active agent may be a promising way to stabilize amorphous materials.
